# Is investigator background related to outcome in head to head trials of psychotherapy and pharmacotherapy for adult depression? A systematic review and meta-analysis

**DOI:** 10.1371/journal.pone.0171654

**Published:** 2017-02-03

**Authors:** Ioana A. Cristea, Claudio Gentili, Pietro Pietrini, Pim Cuijpers

**Affiliations:** 1 Department of Clinical Psychology and Psychotherapy, Babeş-Bolyai University, Cluj-Napoca, Romania; 2 Meta-Research Innovation Center at Stanford (METRICS), Stanford University, Stanford, California, United States of America; 3 Department of General Psychology, University of Padua, Padua, Italy; 4 IMT Institute for Advanced Studies, Lucca, Italy; 5 Department of Clinical Psychology, VU University, Amsterdam, The Netherlands; 6 EMGO Institute for Health and Care Research, Amsterdam, The Netherlands; Universitat Wien, AUSTRIA

## Abstract

**Background:**

The influence of factors related to the background of investigators conducting trials comparing psychotherapy and pharmacotherapy has remained largely unstudied. Specializations emphasizing biological determinants of mental disorders, like psychiatry, might favor pharmacotherapy, while others stressing psychosocial factors, like psychology, could promote psychotherapy. Yet financial conflict of interest (COI) could be a confounding factor as authors with a medical specialization might receive more sponsoring from the pharmaceutical industry.

**Method:**

We conducted a meta-analysis with subgroup and meta-regression analysis examining whether the specialization and affiliation of trial authors were associated to outcomes in the direct comparison of psychotherapy and pharmacotherapy for the acute treatment of depression. Meta-regression analysis also included trial risk of bias and author conflict of interest in relationship to the pharmaceutical industry.

**Results:**

We included 45 trials. In half, the first author was psychologist. The last author was psychiatrist/MD in half of the trials, and a psychologist or statistician/other technical in the rest. Most lead authors had medical affiliations. Subgroup analysis indicated that studies with last authors statisticians favored pharmacotherapy. Univariate analysis showed a negative relationship between the presence of statisticians and outcomes favoring psychotherapy. Multivariate analysis showed that trials including authors with financial COI reported findings more favorable to pharmacotherapy.

**Discussion:**

We report the first detailed overview of the background of authors conducting head to head trials for depression. Trials co-authored by statisticians appear to subtly favor pharmacotherapy. Receiving funding from the industry is more closely related to finding better outcomes for the industry’s elective treatment than are factors related to authors’ background.

**Limitations:**

For a minority of authors we could not retrieve background information. The number of trials was insufficient to evidence subtler effects.

## Introduction

### Conceptual background

Two treatment alternatives dominate the field the field of mental health: psychotherapy and pharmacotherapy. For depression, their effectiveness appears to be similar, with meta-analyses of direct comparisons not finding any differences[1,2]. Previous analyses have mostly focused on examining the effects of factors intrinsic to the trial, such as characteristics of the design, participants, or interventions, on outcomes. However, the influence of factors extrinsic to the trials, such as those related to the *actual* investigators conducting them has largely remained ignored, with the notable exception of investigator funding or financial conflict of interest (COI)[3].

These treatment alternatives are grounded in different conceptions about mental disorders. In the oft-cited bio-psycho-social model of mental disease, psychotherapy emphasizes the psychosocial component, while pharmacotherapy the biological one. The discrepancy leads to conjectures about the individuals conducting trials confronting these treatments, and their own preferred assumptions, which may bias them, even outside awareness, towards favoring one type of intervention over the other. For instance, a researcher assuming that the biological component of mental disorders is essential might unwittingly lean towards seeing medication as a better or more necessary course of treatment. On the other hand, a researcher supposing the psychosocial component is at the root of mental illness might be influenced towards viewing psychotherapy as more useful. Though it would extremely difficult to catalogue the assumptions of all researchers conducting clinical trials, a possible proxy might be looking at the background of trial authors, such as their specialization (i.e., academic degree) or affiliation (i.e., the type of department in which they are professionally active). We would hypothesize that studies where psychiatrists or other medical specializations are in lead author positions or numerically dominate the author pool have a tendency towards finding pharmacotherapy as more effective. Conversely, studies led or dominated by psychologists or other social scientists might systematically find psychotherapy as more effective. Finally, we would expect trials where lead authors are affiliated to medical departments to be partial to treatments approaching mental illness as any other medical condition.

However, authors from other methodological backgrounds, such as statistics, might assume lead positions or be well represented in the author pool. These specializations could be construed as uncontaminated by assumptions regarding the primacy of biological over psychosocial factors in mental disease, and consequently serve as a useful comparison. Recommendations have been made that statisticians and methodologists should be systematically involved in research design and analysis[4], under the assumption that their presence would boost confidence in the quality of the research design, analysis and reporting, and we could assume they employ the “best” methods available. In this sense, investigating whether their presence in the author pool might influence outcomes is particularly germane. Preliminary evidence suggests that studies including statisticians in the research team use a common statistical test more appropriately[5] and that fewer trials are presented as positive when statisticians are listed as co-authors [6].

Nonetheless, some relevant confounding factors could also come into play. Authors with a medical specialization might be more alluring to the pharmaceutical industry and subsequently more sponsored. In turn, this could translate into a biasing effect in favor of medication not due to specialization *per se*, but to side benefits from the industry (i.e., author COI). Trial risk of bias has been consistently related to outcomes both in trials of psychotherapy and pharmacotherapy for depression[7,8]. It is possible the effects of investigators’ preferred assumptions about mental illness and its treatment might be more potent in trials with a higher risk of bias[9].

We were unable to find any previous studies examining whether the background of authors conducting clinical trials that directly compare psychotherapy and pharmacotherapy might have a systematic effect on outcomes. Also, while trials are usually conducted by an assortment of investigators of different backgrounds, we could not retrieve any literature on the composition of this mix or the proportion with which certain fields were represented.

### Study objectives

The first goal was to provide an overview regarding the specialization and affiliation of authors of trials comparing psychotherapy and pharmacotherapy for depression, as well as of the proportions with which medical, social science and statistical/technical academic fields were represented. The second objective involved examining whether these background variables, both applied to the lead authors of the report describing a trial and to the composition of the author pool, could systematically bias the results of the comparison between these two treatments.

## Method

### Identification and selection of studies

Studies were selected from a database of papers on the psychological treatment of depression described in detail elsewhere[10] and that has been used in a series of earlier published meta-analyses (www.evidencebasedpsychotherapies.org). This database is updated at the beginning of each year through comprehensive literature searches. In these searches, abstracts from Pubmed, PsycInfo, Embase and the Cochrane Register of Trials were identified by combining search terms indicative of psychological treatment or psychotherapy and depression (both MeSH terms and text words). The exact search string for Pubmed is provided in S1 File. For this database, primary studies from earlier meta-analyses of psychological treatment for depression were also checked to ensure that no published studies were missed. For this analysis, we used the available version of the database covering studies published between 1966 to January 2015. Two researchers independently performed the selection of the studies from the database.

We included (a) randomized trials (b) in which the effects of a psychotherapy (c) was directly compared with the effects of antidepressant medication (d) in adults (e) with a depressive disorder for outcomes (f) measured on validated symptom scales or diagnostic instruments for depression. All forms of psychotherapy, regardless of their type (e.g., cognitive behavioral therapy, interpersonal therapy), format (individual, group), intensity or duration were included, as well as all forms of antidepressant medication, regardless of their type (e.g., tricyclics, SSRIs), dose or duration of treatment. We excluded augmentation studies in which medication or psychotherapy were combined and compared to one treatment, due to the impossibility of disentangling the effects of psychotherapy and pharmacotherapy in these trials. We also excluded maintenance or continuation studies, focused on patients who had fully or partially recovered from an earlier treatment, again because these trials included add-on effects of psychotherapy and medication. Studies on inpatients were also excluded due to the particular constraints imposed by this setting and the fact that inpatients are medicated most of the time. However, studies where participants had other comorbid mental or somatic disorders in addition to depression were included.

There was no registered protocol for the present systematic review.

### Risk of bias (RoB) assessment and data extraction

#### Assessment of risk of bias

As described in a previous paper[3], trial RoB was rated using four criteria of the Cochrane Collaboration[11] assessment tool: adequate generation of allocation sequence; concealment of allocation to conditions; the prevention of knowledge of the allocated intervention (blinding of assessors); and dealing with incomplete outcome data. Blinding of assessors was rated as low risk if the trial described proper methods of ensuring it or if all relevant outcome measures were self-report, thus not requiring the direct interaction with an assessor. Dealing with incomplete outcome was rated as low risk when intent-to-treat (ITT) analyses, including all randomized patients were conducted, regardless of their subsequent initiation of or participation in treatment. All methods of conducting ITT that included all randomized participants were considered valid, as the Cochrane Collaboration does not make any specific recommendations and many of the trials predate recent discussions regarding the validity of certain methods of handling missing data[12,13]. We also computed a “risk of bias” score for each study, by giving one point to each domain for which a study could be rated as *low* RoB.

#### Extraction and coding of moderators

As we were unable to find any published literature looking at author specialization, we constructed an operational definition. Author *specialization* was defined as the academic field in which the person had graduated or been qualified in and was evaluated for *each* of the authors of the report. However, since in many cases authors have a rich and diverse training, we were constrained to operate with a number of coding decisions that sacrificed granularity. Specialization was coded in the following categories: psychiatry/other medical degree (MDs); psychologist/other social science (e.g., Social Work); Statistics/Epidemiologist/other “purely” technical professions (e.g., Physics, Engineering). Medical doctors were *always* considered in the first category, even if they had a subsequent advanced degree in another field, like Psychology. This decision was based on several considerations. First, a degree in Medicine is an advanced degree associated with cultural assumptions of intellectual prestige (justified or not), as proven by the fact medical doctors almost never fail to include this title (“MD”) together with their name, regardless of whether or not they have obtained additional degrees afterwards. Secondly, this information is certain and lends itself to independent verification as it almost invariably reported by authors. This renders its coding less subjective and more reproducible. For authors who were not MDs, in the case of multiple post-graduate degrees, we considered the one that fit into one of the remaining categories: psychologist/other social science and statistics/epidemiology/other “technical”. In the case in which the authors had multiple degrees, which did not fit exactly fit in any of our categories, the clearest fit to our classification (usually, the first or undergraduate degree) was considered. It should also be noted that due to the diversity of several graduate and post-graduate specializations, these are often harder to classify and information is often times missing about the academic concentration of these programs. For instance, a person with a degree in Psychology and graduate studies in Neuroscience was classified in the psychologist/other social science category. However, depending on the particular program concentration, an advanced degree in Neuroscience could also be considered more medical. Thus, using the graduate degree would have been made the classification of this particular author difficult.

Some types of degrees were considered exceptions and were not counted in any of these categories. Based on our familiarity with the literature on psychotherapies for depression, we anticipated *a priori* that some authors would be trained as nurses and, respectively, economists. While nursing could be seen as a medical degree, nurses are also often trained in psychotherapy[14] and in charge of delivering it in the trial, while medication is still delivered by a psychiatrist. Moreover, different academic systems have different frameworks for education in Nursing, with it being incorporated within the faculty of Medicine in some cases, and it being an independent, stand-alone faculty in others. Consequently, it becomes difficult to establish whether they would, by virtue of their specialization, be more partial to pharmacotherapy or, on the contrary, given their training and role in the trial, to psychotherapy. Biomedical specializations, such as pharmacology, were also difficult to classify and excluded from the classification. These specialists are usually not involved in patient interaction and as such it is unclear whether they would hold *a priori* assumptions about what component (biological or psychosocial) might be more relevant in treatment. Finally, we also did not include degrees in economy in the analysis, since economists do not acquire specialized knowledge about mental disease and its treatment and do not have patient contact. A case could be made for including them in the technical category, but we were worried this might become too heterogeneous if it combined so many different specializations.

**Fig 1:**
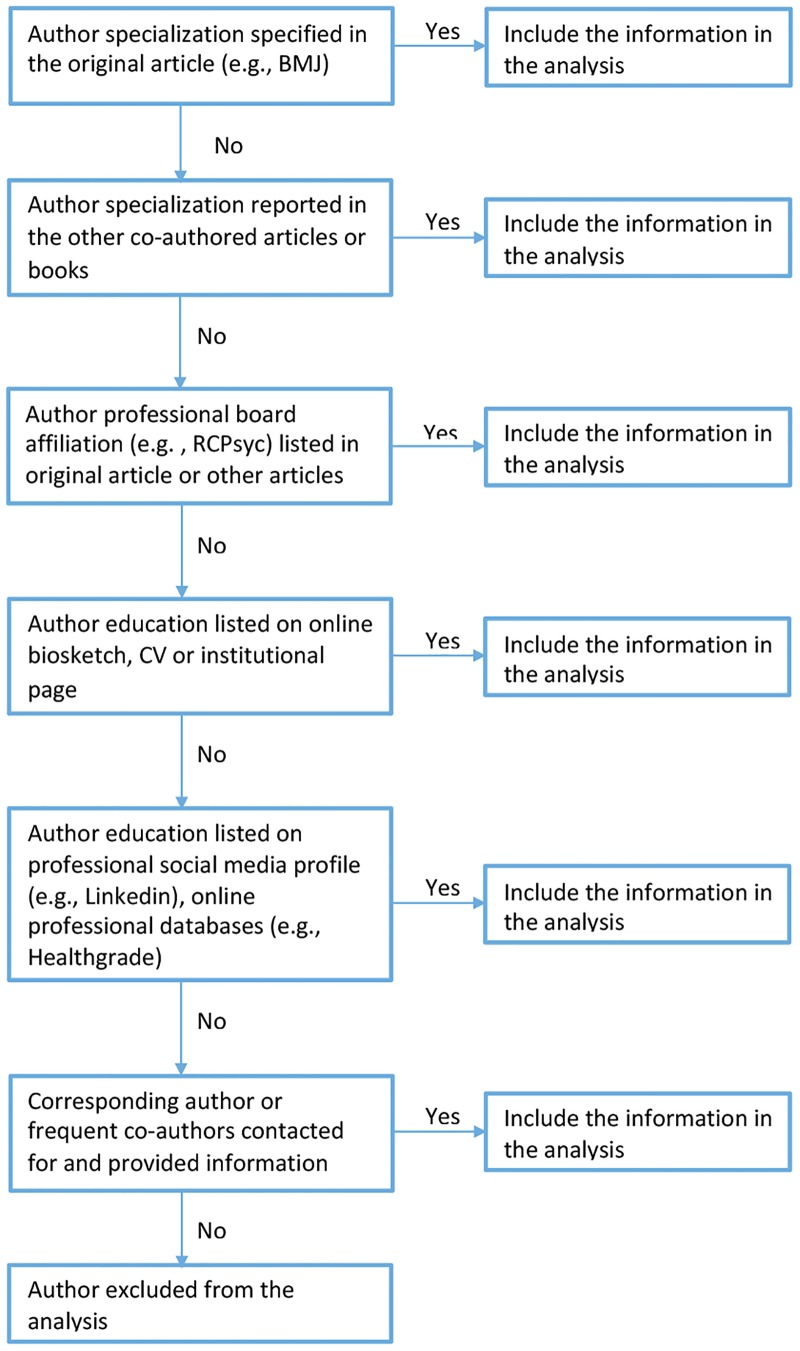
Search and extraction of information about author specialization: Sequential decision tree.

The process of extracting information about author specialization followed a sequential decision tree approach, schematically represented in Fig 1. In this process priority was given to the specialization with which the authors *themselves* identified, since we primarily considered author reported information present in the main paper or in other co-authored works (articles, books).

We *first* examined what was reported in the article, as it is the practice of some journals to also list the authors’ academic specialization (e.g., *The BMJ*, *British Journal of Psychiatry*). If this information was not available, we looked for other articles or book chapters and prefaces by the same author published around the same period (2 years before and after). This choice was made as particularly books include a brief author description usually written by the author herself. Next, we looked as to whether the original article or other articles in the same period included other relevant information, such as affiliations to professional boards like the Royal College of Psychiatrists. In these cases, we checked if the rules of the professional board imposed that members hold a particular type of degree (usually an MD degree for medical professional boards). If we still had not managed to retrieve the information about a particular author’s specialization, we subsequently proceeded to search each author from the report using the search engine Google. We primarily looked for personal or institutional web pages (e.g., university, hospital) or bio sketches available online, as in most cases these include the authors’ educational background. If these were not available, we passed to social network profiles listing academic preparation (e.g., Linkedin, Researchgate), professional databases (e.g., Healthgrades), as well as verified websites that aggregate existent data into professional profiles (www.zoominfo.com). Finally, if no information was available through these extensive internet searches, the corresponding author of the paper was contacted or, if this author was unavailable (usually in the case of old papers), other researchers that were frequent co-authors. The authors for whom we did not manage to find information through any of these methods were excluded from the analyses.

Author *affiliation* was defined as the department in which the author was professionally active (e.g., psychology, psychiatry), regardless of the larger institutional context in which the department was inserted (e.g., medical hospital, national research institution). In the infrequent cases in which the author was not affiliated with a department, the closest similar formal group (e.g., research center, institute) was considered. Affiliation was coded was medical (referring to any medical or biomedical department or similar institute, including but not restricted to psychiatry); non-medical (referring to any type of non-medical department, including psychology, other social sciences or technical departments). In the case of multiple affiliations, priority was given to the medical ones, as with specialization. This coding decision was made because medical affiliations constituted the largest portion of our sample and thus represented a more homogenous category. In contrast, the non-medical category included a vast array of affiliations and research areas. Information about the affiliation was extracted from the reports, as were interested at the authors’ affiliation at the time the trial was conducted and published.

Data regarding specialization and affiliation was organized in a number of categorical and continuous variables both regarding lead authors (first and last) and the composition of the author pool:

1. Specialization of the first and respectively last author;
2. Affiliation of the first and last author;
3. The proportions, expressed in percentages, from the total number of authors of a trial for: psychologists; social scientists (psychologists plus other degrees); psychiatrists; MDs (psychiatrists plus other MDs) and respectively, statisticians/epidemiologists/other technical specialty.
4. The ratio of the number of psychologists to the number of psychiatrists, and respectively social scientists to MDs.

We also coded *author financial conflict of interest* (COI), in order to include in multivariate analyses. Author COI was the subject of a more detailed previous analysis on this dataset, which includes more extensive information about its evaluation[3]. Briefly put, it was defined as the receipt of financial support or benefits of any type from the industry. Information about COI was extracted primarily from the articles included in the meta-analysis, but also from other reports co-authored by the same individuals and originating in the same time frame. This variable was coded dichotomically for each trial into: yes (at least one of the authors of the report has COI) and not reported (no information about COI). We did not include a separate category of no COI (i.e., authors having declared not having any COI) because our previous analysis on these trials showed these declarations can often be inaccurate[3]. In it, we identified instances in which one or more of the authors of the original article had a financial COI and had not reported it in the main article. Moreover, the number of cases in which the authors explicitly declared not having any financial COI was very small.

Data regarding specialization, affiliation, and author COI was extracted from the papers and the additional resources described above by one researcher. Subsequently, a third of the included papers was randomly chosen (using a random number generator) for independent evaluation by another researcher, who did not have access to the data extracted by the first. Risk of bias ratings were carried out by two independent researchers. Disagreements were resolved through discussion.

### Meta-analysis

#### Effect size calculation

We calculated and pooled the individual effect sizes with the computer program Comprehensive Meta-analysis (CMA; version 3.3.070), using a random effects meta-analysis. For each comparison between psychotherapy and pharmacotherapy, an effect size (ES) indicating the difference between the two groups at post-test (Hedges' *g*) was calculated by subtracting the mean of the pharmacotherapy group from the mean of the psychotherapy group, dividing the result by the pooled standard deviation, and correcting for small sample bias[15]. In the cases where a study included more than one comparison between a form of psychotherapy and a drug, we averaged comparisons at study level employing the procedures implemented in CMA[16]. Sensitivity analyses were also conducted using only the comparisons with the ES most favourable to psychotherapy and, respectively, pharmacotherapy. For the studies that only reported data in subgroups (and not for the overall psychotherapy or pharmacotherapy group), subgroups were averaged at the study level. Outcomes were considered only if they were measured with instruments explicitly validated for depressive symptoms, such as Hamilton Rating Scale for Depression (HAM-D) or the Beck Depression Inventory (BDI). For studies where means and standard deviations were not reported and could not be obtained from the authors, we transformed dichotomous data into the standardized mean difference using the formulas implemented in CMA[16] or used other statistics, such as *t*-values or exact *p*-values to calculated the standardized mean difference. We calculated the *I*^*2*^-statistic as an indicator of heterogeneity in percentages. A value of 0% indicates no observed heterogeneity, while larger values indicate increasing heterogeneity, with 25% as low, 50% as moderate, and 75% as high heterogeneity[17]. We calculated 95% confidence intervals (CIs) around *I*^*2*^ [18], using the non-central χ^2^-based approach with the *heterogi* module for STATA[19]. Outliers were defined as ESs for which the 95% CI was outside the 95% CI of the pooled studies.

#### Publication bias

Publication bias was assessed in three ways: 1) visual inspection of the asymmetry of the funnel plot; 2) the Duval-Tweedie trim and fill procedure[20] (as implemented in CMA, version 3.3.070, searching for missing studies left of the mean using a fixed effects model), which provides an adjusted effect size after the publication bias has been taken into account and also indicates how many studies were imputed to correct for publication bias; and 3) Egger’s test of the intercept to test the symmetry of the funnel plot[21].

#### Subgroup and meta-regression analysis

The focus on our analysis was on subgroup and meta-regression analysis. For subgroup analyses, we used a mixed effects model[16] to test whether there were differences among studies regarding first and last author specialization and affiliation (coded categorically). We conducted both univariate and multivariate analyses using the same program, CMA version 3.3.070. Meta-regression analyses were conducted according to a random effects model using the Knapp-Hartung method. Univariate analyses were conducted separately for variables regarding the percentage of social scientists, MDs, and respectively, statisticians /epidemiologist/other technical specialty from the total number of authors. To reduce the number of statistical tests we only used the most inclusive category (i.e., social scientists and respectively MDs). The ratio of psychologists to psychiatrists, or social scientists to MDs, was not used in subgroup and meta-regression analyses. This was due to the fact that in part this variable duplicated information already existent in other indexes considered (i.e., proportion of psychologists or psychiatrists) and also to its highly skewed distribution, with extreme values at both ends. This asymmetry raised doubts about the stability of any results using this variable, as they could be driven by extreme values.

Multivariate analysis included specialization and affiliation as predictors of interest, together with RoB score and author COI. Categorical variables were dummy coded for use in multivariate analysis, with one category being used as the reference category. The medical category was used as the reference category for both specialization and affiliation, as it was constructed based on the most certain, reliable information, and because the contrast with medical specializations and affiliations was one of our main points of interest.

In order to avoid collinearity, for variables that correlated with an r greater than 0.50, we just included one in the analyses. Prior to conducting multivariate analysis, we computed correlations between predictors of interest. Multivariate analysis also included the more inclusive categories of percentages of authors social scientists, and respectively MDs.

## Results

### Selection and inclusion of studies

**Fig 2:**
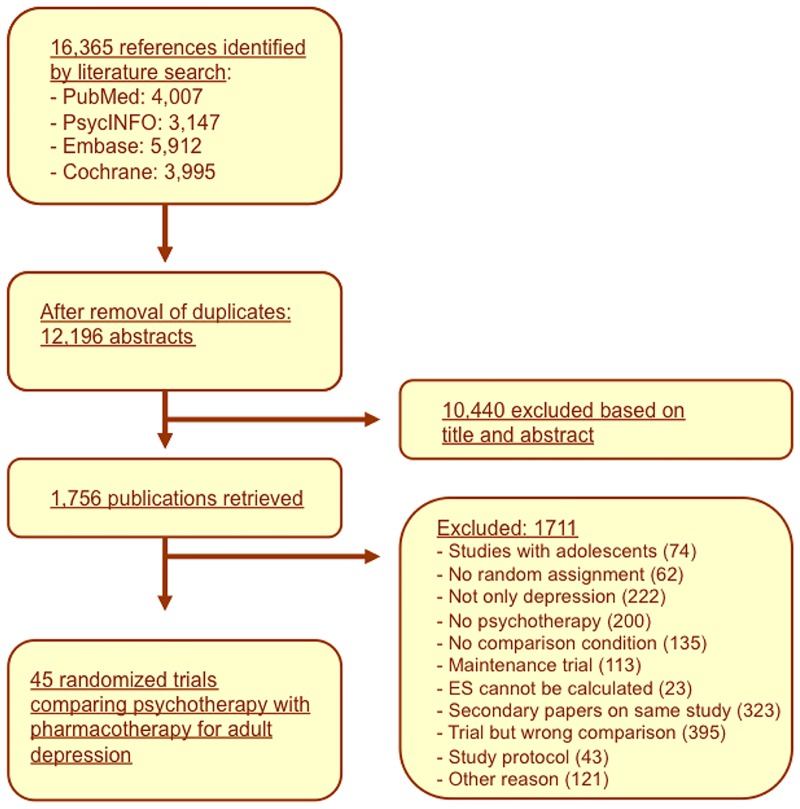
Flowchart of selection and inclusion process, following the PRISMA statement.

A total of 16,365 abstracts (12,196 after removal of duplicates) was examined and 1,756 full-text papers were retrieved for detailed consideration. The PRISMA flowchart describing the inclusion process is presented in Fig 2. We excluded 1,711 records from the retrieved papers (reasons given in the flowchart). Forty-five trials met the inclusion criteria (full study list in S2 File). Out of these, nine included 2 comparisons between a form of psychotherapy and medication.

### Characteristics of the included studies

Selected characteristics of included studies focused on the variables of interest are present in Table 1. We note that a more detailed description of included studies in terms of participants, interventions and other trial features can be found in previous papers on this database[1].

**Table 1:** Selected characteristics of studies directly comparing psychotherapy and pharmacotherapy for adult depression.

Study	1^st^ AU Spec	1^st^ AU Affil	Last AU Spec	Last AU Affil	%Psychol	%Soc Sci	%Psyc	%MD	%Stat/Techn	Psychol/Psych	Soc Sci/MD	COI	RoB^a)^ SG AG BA ITT
Barber, 2011	Psychol	Med	Psych	Med	40	40	40	40	20	1	1	Y	+ ? + +
Barrett, 2001	Psych	Med	Stat	Med	33	33	22	56	11	1.5	0.6	Y	+ ? + −
Bedi, 2000	Psych	Med	MD	Med	0	0	36	55	27	0	0	NR	? + SR −
Blackburn, 1997	Psychol	Non-med	Psychol	Non-med	100	100	0	0	0	Indet	Indet	NR	? ? + −
Blom, 2007	Psych	Med	Psych	Med	29	29	43	43	14	0.6	0.6	NR	? ? + −
Browne, 2002	Nurse	Non-med	MD	Med	0	0	0	55	0	0	0	NR	+ + + −
David, 2008	Psychol	Non-med	Psych	Med	50	50	50	50	0	1	1	NR	? ? + +
Dekker, 2008	Psychol	Non-med	Psych	Med	44	44	56	56	0	0.8	0.8	NR	? ? + −
DeRubeis, 2005	Psychol	Non-med	Stat	Non-med	55	55	36	36	9	1.5	1.5	NR	? ? + +
Dimidjian, 2006	Psychol	Non-med	Psychol	Non-med	85	85	8	8	8	11	11	Y	+ − + +
Dunlop, 2012	Psych	Med	MD	Med	50	50	17	33	17	3	1.5	Y	? + + −
Dunner, 1996	Psych	Med	NI	Med	50	50	33	33	0	1.5	1.5	NR	? ? + −
Elkin, 1989	Psychol	Non-med	Psychol	Non-med	67	67	25	25	8	2.6	2.6	NR	+ ? + −
Faramarzi, 2008	Psychol	Med	NI	Med	50	50	17	33	0	3	1.5	NR	? ? SR −
Finkenzeller, 2009	MD	Med	Psych	Med	40	40	20	60	0	2	0.7	NR	+ ? + +
Frank, 2011	Psychol	Med	Econ	Med	13	13	40	40	33	0.3	0.3	Y	− − + +
Hegerl, 2010	Psych	Med	Psych	Med	56	56	33	44	0	1.7	1.2	Y	+ + + +
Hollon, 1992	Psychol	Non-med	Psych	Med	71	71	29	29	0	2.5	2.5	NR	? ? + +
Jarrett, 1999	Psychol	Med	Stat	Med	50	50	17	17	33	3	3	NR	? + + +
Keller, 2000	Psych	Med	Psych	Med	25	25	67	75	0	0.4	0.3	Y	+ + + −
Kennedy, 2007	Psych	Med	MD	Med	43	43	29	43	0	1.5	1	Y	? ? ? −
Markowitz, 2005	Psych	Med	Psych	Med	20	20	60	60	20	0.3	0.3	Y	+ − + +
Martin, 2001	Psych	Med	Eng	Med	0	0	40	40	40	0	0	Y	− − − +
McKnight, 1992	Psychol	Med	Psych	Med	67	67	33	33	0	2	2	NR	? ? ? −
McLean, 1979	Psychol	Med	Psychol	Non-med	100	100	0	0	0	Indet	Indet	NR	? ? SR −
Menchetti, 2014	Psych	Med	Psych	Med	0	0	71	71	29	0	0	Y	+ + ? +
Miranda, 2003	Psychol	Med	Stat	Non-med	57	57	14	14	29	4	4	NR	+ + + +
Mohr, 2001	Psychol	Med	Psych	Med	40	40	20	40	20	2	1	NR	− − − +
Moradvesi, 2013	Psychol	Non-med	Psychol	Non-med	80	80	20	20	0	4	4	NR	+ + + +
Murphy, 1984	Psych	Med	Psychol	Med	75	75	25	25	0	3	3	NR	− − − −
Mynors-Wallis, 1995	Psych	Med	MD	Med	0	0	50	100	0	0	0	NR	? + + −
Mynors-Wallis, 2000	Psych	Med	MD	Med	0	0	50	75	0	0	0	NR	+ + + +
Parker, 2013	Psych	Med	Psychol	Med	64	64	27	27	0	2.3	2.3	Y	+ + + −
Quilty, 2008	Psychol	Med	Psychol	Med	100	100	0	0	0	Indet	Indet	NR	? ? ? −
Rush, 1977	Psych	Med	Psychol	Med	50	50	50	50	0	1	1	NR	? ? + −
Salminen, 2008	MD	Non-med	Stat	Non-med	13	13	63	75	13	0.2	0.1	Y	? ? − +
Schulberg, 1996	Psychol	Med	MD	Med	20	30	10	40	10	2	0.7	NR	? ? + +
Scott, 1992	Psych	Med	Psych	Med	0	0	100	100	0	0	0	NR	? + + −
Shamsaei, 2008	Nurse	Med	Stat	Med	25	25	25	25	25	1	1	NR	? + SR ?
Sharp, 2010	MD	Med	Stat	Med	0	0	33	58	8	0	0	Y	+ + + +
Sloane, 1985	MD	NI	Psych	NI	33	33	67	67	0	0.5	0.5	NR	− − + −
Thompson, 2001	Psychol	Med	MD	Med	60	60	20	40	0	3	1.5	NR	? ? ? −
Weissman, 1979	Soc work	Med	Psych	Med	0	17	67	67	0	0	0.2	NR	? ? + −
Williams, 2000	MD	Med	Stat	Med	38	38	13	50	13	3	0.7	Y	+ + + +
Zu, 2014	MD	Med	MD	Med	0	0	15	85	0	0	0	NR	+ ? + −

Note.

^a)^ RoB: risk of bias according to the Cochrane Collaboration tool. SG, sequence generation; AC, allocation concealment; BA; blinding of assessors; ITT, intent-to-treat analysis to handle missing data. Ratings of “+” indicate the study has a low RoB on that criteria; ratings of “-”indicate high RoB; “?” uncertain RoB; SR, only self-report measures

Abbreviations: Affil, Affiliation; AU, Author; COI, Conflict of interest; Econ, Economist; Eng, Engineer; Indet, Indeterminate; MD, Medical doctor; Med, Medical; NI, no information; Non-med, Non-medical; NR, not reported; Psych, Psychiatrist; Psychol, Psychologist; Spec, specialization; Soc Sci, Social scientist; Soc work, Social work; Stat, Statistician; Techn, Technical; Y, yes.

The first author was a psychologist or social scientist in 20 studies (in 19 cases with a degree in psychology, in one with a degree in social work), a psychiatrist or MD in 23 studies (17 psychiatrists and 6 other MDs) and a nurse in 2 studies. The last author was a psychologist in 9 studies, an economist in 1, a psychiatrist in 15, an MD of another kind in 9, a statistician/ epidemiologist/other technical in 9 studies. For 2 studies we could not retrieve information about the last author. Affiliation was mostly medical both for the first author (34 trials) and for the last author (37 trials). The composition of the author pool was variable. Three reports were authored exclusively by psychologists, and 10 included no psychologists at all. In the remaining trials, the ratio of psychologists to psychiatrists ranged from 0.2 to 11, and 9 studies had three times more psychologists than psychiatrists. Twenty-four studies included no authors from the category statisticians/epidemiologists/other technical. Author COI was present in 15 trials, while for the other 30 it was not reported and we could not uncover additional information pointing to it.

### Meta-analysis

#### Publication bias

Similar to what was reported in a previous papers[3] on this database of trials and the comparison between psychotherapy and pharmacotherapy, there was evidence of publication bias in the visual inspection of the funnel plot. The Duval-Tweedie trim and fill procedure imputed 6 studies, leading to a pooled ES more favorable to pharmacotherapy but still not significant, g = -0.11, 95% CI -0.22 to 0.003. However, Egger’s test did not indicate a significant asymmetry of the funnel plot (intercept = 0.91, 95% CI -0.34 to 2.16).

#### Subgroup analyses

Subgroup analyses (Table 2) indicated a significant difference for the specialization of the last author (p = 0.002). Specifically, studies where the last author was classified as statistician/epidemiologist/other technical found a small statistically significant advantage of pharmacotherapy over psychotherapy (*g* = -0.25, 95% CI -0.37 to -0.14). Studies where the last author was either psychologist/social scientist or psychiatrist/MD did not find significant differences between the two treatments. To further explore this effect, we also classified the proportion (expressed as percentages) of statisticians/epidemiologists/other technical from the total author pool in: none; over 0 and under 20%; 20% or more. There were significant differences between these 3 subgroups (p = 0.031). Trials with at least one author but less than 20% from this category resulted in a pooled ES favoring pharmacotherapy over psychotherapy (*g* = -0.14, 95% CI -0.26 to -0.01). Differences between the two treatments were not significant in studies with more than 20% of authors from this category. No other subgroup comparisons were statistically significant.

**Table 2:** Effects of studies comparing psychotherapy and pharmacotherapy for adult depression^a^.

Variable	n_comp_	*g*	95% CI	*I*^*2*^	*I*^*2*^ 95% CI	p^b^	Slope	95% CI	p
**Depression**	45	-0.02	-0.12~0.07	58	38~69				
One ES per study (most favourable to psychotherapy) ^c^	45	-0.003	-0.10~0.10	60	42~71				
One ES per study (most favourable to pharmacotherapy) ^c^	45	-0.04	-0.14~0.06	57	38~69				
Outliers removed^d^	41	-0.07	-0.14~0.004	22	0~47				
**Subgroup analysis**^e^									
1^st^ Author Specialization: Psychologist/Social scientist	20	0.009	-0.15~0.16	63	34~76	0.07			
Psychiatrist/MD	23	-0.01	-0.15~0.12	53	1~66				
Nurse	2	-0.31	-0.55~-0.07	0	n/a^f^				
1^st^ Author Affiliation^g^: Non-medical	10	-0.005	-0.23~0.22	67	23~82	0.85			
Medical	34	-0.03	-0.14~0.08	56	32~70				
**Last Author Specialization**^h^: Psychologist/Social scientist	8	0.12	-0.17~0.40	60	0~80	**0.002**			
Psychiatrist/MD	25	0.02	-0.10~0.15	52	15~68				
**Statistician/Technical**	**9**	**-0.25**	**-0.37~-0.14**	**0**	**0~54**				
Last Author Affiliation^g^: Non-medical	7	0.07	-0.24~0.38	76	38~87	0.49			
Medical	37	-0.04	-0.15~0.06	54	28~67				
**Proportion of statisticians**: None	24	0.11	-0.05~0.27	64	39~76	**0.031**			
**> 0 and < 20%**	**11**	**-0.14**	**-0.26~-0.01**	**22**	**0~66**				
20% or more	10	-0.17	-0.05~0.27	53	0~75				
% Social Scientists							0.002	-0.002~0.006	0.30
% MDs							0.001	-0.004~0.006	0.63
**% Statisticians/Technical**							**-0.01**	**-0.02~-0.0004**	**0.041**

Note.

^a^ All results are reported with Hedges ***g***, using a random effects model. A positive effect indicates superiority of psychotherapy.

^b^ The *p* levels in this column indicate whether the difference between the ESs in the subgroups is significant (significant results are marked with bold)

^c^ Studies with more than one comparison: David, 2008; Dimidjian, 2006; Elkin, 1989; Markowitz, 2005; McLean, 1979; Mohr, 2001; Mynor-Wallis, 2000; Quilty, 2008; Scott, 1992.

^d^ Outliers were defined as studies in which the 95% CI was outside the 95% CI of the pooled studies. Above the 95% CI (favoring psychotherapy): Faramarzi, 2008; Moradveisi, 2013; Rush, 1977. Below the 95% CI (favoring pharmacotherapy): Sharp, 2010

^e^ Subgroup analysis were conducted using a mixed effects model.

^f^ Confidence intervals around *I*^*2*^ cannot be calculated if there are less than 3 groups

^g^ Sloane et al., 1985 did not report information on the affiliation of the author

^h^ We were unable to retrieve information about the specialization of the last author of Dunner et al., 1996 and Faramarzi et al., 2008. In another study (Frank et al., 2011), the last author was an economist

#### Univariate meta-regression

Univariate analysis (Table 2) indicated a significant negative relationship between the proportion (expressed as percentages out of the total number of authors) of authors statisticians/epidemiologists/other technical and outcomes in favor of psychotherapy (slope coefficient b = -0.01, 95% CI -0.02 to -0.0004, p = 0.041). In other words, an increase in the proportion of authors from this category was associated with better outcomes for pharmacotherapy over psychotherapy. The relationships between the proportion of psychologists/social scientists, and respectively psychiatrists/MDs, with depression outcomes were not significant.

#### Multivariate meta-regression

We examined two multivariate models, both detailed in Table 3. First author specialization was dummy coded into one variable examining the contrast between psychologists/social scientists and psychiatrists/MDs. Last author specialization was coded into two dummy variables, corresponding to the contracts between psychologists/social sciences and respectively statisticians/technical as contrasted to psychiatrists/MDs. Affiliation was dummy coded into medical versus non-medical, and author financial COI into existent versus not reported.

**Table 3:** Multivariate meta-regression analyses with all moderators of interest.

Depression (all outcomes)	Multivariate: Full model		
	Coeff	95% CI	p ^a)^
**Model a**			
RoB	0.05	-0.04~0.15	0.25
**1**^**st**^ **Author Specialization Psychologist/Social scientist vs Psychiatrist/MD**	**-0.25**	**-0.51~0.01**	**0.06**
Last author Specialization: Psychologist/Social scientist vs Psychiatrist/MD	0.21	-0.10~0.52	0.17
**Last Author Specialization: Statistician/Technical vs Psychiatrist/MD**	**-0.26**	**-0.53~0.01**	**0.06**
Last Author Affiliation: Non-medical vs Medical	0.13	-0.20~0.47	0.43
**Author Financial COI: Present vs Not reported**	**-0.27**	**-0.53~-0.01**	**0.04**
**Model b**			
RoB	0.008	-0.09~0.11	0.87
Last Author Affiliation: Non-medical vs Medical	0.13	-0.19~0.45	0.42
% MDs	0.002	-0.003~0.007	0.44
% Statistician/Technical	-0.008	-0.02~0.003	0.14
Author Financial COI: Present vs not reported	-0.15	-0.41~0.10	0.24

Note.

^a)^ The *p* levels in this column indicate whether the relationship between the moderator and effects sizes is significant in meta-regression analyses (significant results are in bold). Positive coefficients indicate a superiority of psychotherapy.

Coeff, Coefficient; MD, Medical doctor; COI, conflict of interest

Some variables were found to be strongly correlated with each other, and thus we ran separate analyses in which only one was retained. The affiliation of the 1^st^ author correlated with that of the last author (r = 0.59). The percentage of psychologists/social scientists had correlations that exceeded our threshold with the specialization of the 1^st^ author (r = 0.55), the percentage of psychiatrists/MDs (r = -0.78) and with one of the dummy variables for last author specialization that contrasted authors psychologists/social scientists with authors statisticians/other technical (r = 0.66). Additionally, the percentage of author psychiatrists/MDs also had correlations larger than 0.5 with the specialization of the 1^st^ author (r = -0.57), as well as with the same dummy variables for last author specialization (r = -0.51). The percentage of statisticians/other technical was also correlated with the other dummy variable for last author specialization (r = 0.57). Neither risk of bias nor author COI displayed correlations over 0.50 with any of the variables considered. To tackle with the high correlations between some of our predictors of interest, we analyzed two multivariate models: (a) including only last author affiliation, 1^st^ and respectively last author specialization, along with RoB and author COI; (b) including only last author affiliation, percentage of psychiatrists/MDs, and percentage of statisticians/other technical, along with RoB and author COI.

In model a that included 1^st^ and last author specialization and affiliation, we found author financial COI to be a significant predictor (b = -0.27, 95% CI -0.53 to -0.01, p = 0.04). However, we found a close to significance trend for trials where the first author was a psychologist/social scientist to report more favorable outcomes for pharmacotherapy than studies where the first author was a psychiatrist/MD (b = -0.25, 95% CI -.51 to 0.01, p = 0.06). In a similar vein, trials where the last author was a statistician/other technical as contrasted to those where he/she was a psychiatrist/MD showed a borderline significant trend of favoring pharmacotherapy (b = -0.26, 95% CI -0.53 to 0.01, p = 0.06). In model b, which included the proportions of MDs and authors statisticians/other technical, none of the predictors were significantly related to outcomes.

## Discussion

### Summary of main results

Our investigation was based on the notion that the principal treatments for mental disorders -psychotherapy and pharmacotherapy- are grounded in different conceptions of what causes mental disease and which components should be at the center of treatment. Based on this distinction, we queried whether the preferred assumptions of investigators conducting trials comparing these two treatments might bias them, even unwittingly, towards one intervention or another. We conjectured the background of trial authors, as reflected in their specialization and affiliation, might function as a proxy for these assumptions.

The first goal of our study was to provide a more detailed overview regarding the background of investigators who conducted the studies confronting these two treatments for depression. These aspects have remained largely unknown and indeed not considered so far. For the lead authors, our results showed a balanced situation for the specialization of the first author, with about half of the studies having a psychologist and the other a psychiatrist/MD in this position. However, the majority (roughly half) of studies had an author who was a psychiatrist/MD as the last author, with the other half equally split between an investigator psychologist/social scientist or statistician/other technical. Lead authors mostly came from medical settings. The composition of the author pool was variable, but an interesting aspect was the fact that about a half of the studies also had authors from the statistics/epidemiology/other technical category. At a descriptive level, our results offer a useful inventory of the background of investigators of head to head trials of psychotherapy and pharmacotherapy for depression and could be used as a starting point in other studies.

Our second objective was examining potential systematic associations between these background variables and depression outcomes. Subgroup and univariate analyses consistently revealed a pattern regarding authors that were statistician/epidemiologists/other technical professions. Studies in which these authors were in lead positions (last authors) found significantly more favorable results for pharmacotherapy. Also, when looking at the proportion of authors in this category from the total author pool, univariate meta-regression indicated that an increase in this proportion was also associated with better outcomes for pharmacotherapy. In the same vein, subgroup analyses indicated significant differences between studies with varying proportions of authors statisticians/epidemiologists/other technical. More exactly, studies with at least one but less than one fifth of the investigators from this category found more favorable results for pharmacotherapy. Multivariate analysis also showed a borderline significant trend of studies finding more favorable outcomes for pharmacotherapy when the last author was a statistician/other technical as compared to a psychiatrist/MD.

### Implications

We presumed the category of statisticians to be less contaminated by assumptions regarding the primate of biological or psychosocial factors in mental diseases and their treatments. We cited previous evidence in this sense, showing that the presence of statisticians in the author pool is associated with a more appropriate use of a standard statistical test[5] and with less reporting of trials as positive[6]. However, both cited studies used methods of assessing author background that were very different from ours. In the first[5], the authors used a rather crude method of individuating statisticians, considering as such any authors listed as members of departments of statistics, clinical epidemiology and related disciplines, as well as any authors credited for statistical review in the analyses. In comparison, the method we employed brings additional precision and reliability as we checked every individual author. In the other analysis[6], the authors accounted for the presence of statisticians in the author pool by surveying the corresponding author of the trial. This method also has several limitations, as authors might be biased toward reporting that statisticians were present, could use different definitions of statisticians, equate a wide range of specializations with statisticians or conflate proper training with short courses or being an expert by virtue of practice.

One interesting finding that emerged from our analysis was that trials where these authors are better represented or take leading positions arrive to results favoring pharmacotherapy. We can only speculate on the reasons for these results. For instance, since psychologists and social scientists in general usually also receive training in statistics and are more confident in analyzing data, it is conceivable trials where they are dominant don’t usually see the need for statisticians. On the other hand, studies more dominated by the medical profession might be more keen to having statisticians or other such professions on board. However, it is worth noting that the effect was small in magnitude and only borderline significant in multivariate analysis. Given that the presence of statisticians or other such professions could be viewed as providing additional reassurance in the quality of data analysis, with recommendations having been made in this sense[4], these results may imply that pharmacotherapy has somewhat better outcomes than psychotherapy. This idea is also consistent with the fact that adjustment for publication bias shifted the pooled comparative effect size as more favorable to pharmacotherapy than psychotherapy (albeit still non-significant). Yet it is also very well possible these may describe spurious findings, especially since in the multivariate analyses most of these variables were not consistently significant anymore.

For multivariate analyses, given that many of our variables of interest were correlated and their use in the same analysis would have provided biased estimated, we tested two separate models. In the first, we only included affiliation and specialization, while in the second we focused on the proportion of authors MDs and respectively statisticians. In the first model, we found that only author financial COI, defined as authors’ financial ties with the pharmaceutical industry, to be a significant predictor of outcome. Its presence was unsurprisingly associated with better outcomes for pharmacotherapy. We note that this factor was also the subject of a previous more detailed investigation focused on sponsorship bias[3], where we did not consider author background variables, but showed the same trend of studies authored by researchers financially supported by the industry to favoring pharmacotherapy over psychotherapy. In the present analysis, author COI remained a significant predictor even when other variables regarding the specialization and affiliation of study authors were taken into account. We hypothesized that, as investigators, MDs in general and psychiatrists in particular might be more alluring to the pharmaceutical industry and hence benefit from more sponsorship. Consequently, it would seem that receiving funding from the industry is more closely related to finding better outcomes for the industry’s elective treatment than factors having to do with the author background. However, we note that in the second model, where we considered proportions of authors with a psychiatry/MD or statistical/technical specialization, author financial COI was no longer a significant predictor of outcome.

Regarding background variables, the specialization of the first author and respectively last author both came close to statistical significance in the first model. Studies in which the first author was psychologist/social scientist as compared to psychiatrist/MD, and respectively studies where the last author came from the statistical/technical category as compared to a psychiatrist/MD showed a non-significant trend favoring pharmacotherapy over psychotherapy. We note that the first author was a non-psychologist social scientist in only one case, so this result could be construed as referring to the comparison between psychologists and medical doctors. Assuming that psychologists would be inclined, by virtue of training, to view psycho-social factors as essential in the treatment of mental illness, we would have expected them to be biased towards psychotherapy. Yet it is also plausible that in influential publications such as randomized trials for depression, where a large number of contributors is usually involved, the choice of the first author is strategic (e.g., giving visibility to a young researcher) and intertwined with other features of the trial. It is unclear how much leverage the first author of such a trial would actually have in influencing trial outcomes or their presentation. We could speculate this result might also reflect other, unknown, particularities of trials where the first author is a psychologist and that these particularities might be the ones responsible for results favoring pharmacotherapy over psychotherapy. A similar argument probably holds for the result regarding last author specialization. We note there were only 9 trials with a last author statistician/other technical and hence this finding might reflect some distinct features of this pool of studies. It is also possible that both these borderline significant relationships reflect spurious findings.

### Limitations

There are number of limitations to our evaluation. We could not retrieve any previous literature attempting to evaluate author background from trial reports, and as such we could not base our coding decisions on existent literature. To be able to classify extracted data, we were compelled to resort to some simplifications and invariably sacrifice detail and granularity. Evidently, arguments could be given for other coding choices. Information about author specialization was the result of extensive web searches and it is possible we have missed or misinterpreted some potential sources. We tried to be as conservative as possible and only classify authors in one category when we were able to come across precise information about their academic education. This led to another limitation, namely that for a minority of authors we were not able to recover this information. These authors were not included in the analyses and could have potentially altered some of our results. The number of trials comparing psychotherapy and pharmacotherapy for depression, while considerable, might have still been insufficient for revealing more subtle effects of some author background variables. Finally, we only considered financial COI in relationship to the pharmaceutical industry, and not to psychotherapy (e.g., royalties from manuals or books, payment from workshop, courses or training). Unfortunately, even though several red flags have been raised regarding the relevance and need of disclosing and analyzing COI in regards to psychotherapy, the information necessary for its assessment is still lacking[22], and there is still no consensus at to what should be considered under this label. Apart from financial COIs, more intellectual COIs related to the practice of psychotherapy, such the investigators’ commitment and faith in the intervention (i.e., researcher allegiance), or related possible self-confirmatory academic biases (i.e., authors of previous positive trials would be committed to report more positive effects) might also have biasing effects on the comparison between psychotherapy and pharmacotherapy.

### Conclusions

Our study is, to our knowledge, the first to take on the painstaking task of describing and classifying the background of authors of reports of clinical trials for depression. We believe this detailed overview of author background, as well as some of our coding choices, can be resources for future research. In sum, our findings revealed some systematic trends regarding the background of authors conducting trials comparing psychotherapy and pharmacotherapy for depression. The presence of statisticians, particularly in lead positions (last author) appears to favor pharmacotherapy. There is a similar trend for having the first author psychologist as compared to MD. None of these results were anticipated and might reflect spurious findings; as such, speculating on their meaning might not be very relevant. It is worth pointing out these differences are small, in some cases only borderline statistically significant and most likely not clinically significant. Given that psychotherapy and pharmacotherapy were consistently shown to be equally effective in the treatment of acute depression[1,2], it would have been surprising to find anything other than subtle, small magnitude differences. Nevertheless, one factor that does seem to override variables related to the specialization and affiliation of trial authors is financial support from the pharmaceutical industry.

## Supporting information

S1 File Search string and list of included studies. (DOCX)

S2 File List of studies included in the meta-analysis. (DOCX)

S3 File PRISMA checklist. (DOCX)
