# Supplementary material for: Is investigator background related to outcome in head to head trials of psychotherapy and pharmacotherapy for adult depression? A systematic review and meta-analysis
Source: PLoS One. 2017 Feb 3;12(2):e0171654. doi: 10.1371/journal.pone.0171654 (PMC5291442; doi:10.1371/journal.pone.0171654)
Supplement: S1 File — (DOCX) [file pone.0171654.s001.docx]

**S1 File: Search string and list of included studies**

1. Complete search string for Pubmed

("psychotherapy"[MeSH Terms] OR "psychotherap*"[All Fields] OR cbt[All Fields] OR "cognitive behavior* therap*"[All Fields] OR "cognitive behavior* therap*"[All Fields] OR "behavior* therap*"[All Fields] OR "behavior* therap*"[All Fields] OR "cognition therap*"[All Fields] OR psychodynamic[All Fields] OR "psychoanalysis"[MeSH Terms] OR "psychoanalysis"[All Fields]) OR psychoanalytic*[All Fields] OR "counselling"[All Fields] OR "counseling"[MeSH Terms] OR "counseling"[All Fields]) OR "problem-solving"[All Fields] OR "problem solving"[All Fields] OR "mindfulness"[All Fields] OR (acceptance[All Fields] AND  "commitment"[All Fields]) OR "assertiveness training"[All Fields] OR "behavior* activation"[All Fields] OR "cognitive therap*"[All Fields] OR "cognitive restructuring"[All Fields] OR "metacognitive therap*"[All Fields] OR "solution-focused therap*"[All Fields] OR "self-control therap*"[All Fields] OR "self control therap*"[All Fields] OR "self-control training"[All Fields] OR "self-control training"[All Fields])

AND

("depressive disorder"[MeSH Terms] OR ("depressive"[All Fields] AND "disorder"[All Fields]) OR "depressive disorder"[All Fields] OR "depression"[All Fields] OR "depression"[MeSH Terms]) OR depressive[All Fields] OR "major depression"[All Fields] OR "major depressive disorder"[All Fields] OR "dysthymic disorder"[MeSH Terms] OR  ("dysthymic"[All Fields] AND "disorder"[All Fields]) OR "dysthymic disorder"[All Fields] OR "dysthymia"[All Fields] OR dysthymic[All Fields] OR "mood disorder"[All Fields] OR "affective disorder"[All Fields])

Filters: Randomized Controlled Trial
