# Supplementary material for: Is investigator background related to outcome in head to head trials of psychotherapy and pharmacotherapy for adult depression? A systematic review and meta-analysis
Source: PLoS One. 2017 Feb 3;12(2):e0171654. doi: 10.1371/journal.pone.0171654 (PMC5291442; doi:10.1371/journal.pone.0171654)
Supplement: S2 File — (DOCX) [file pone.0171654.s002.docx]

S2 File. List of studies included in the meta-analysis

1

Barber JP, Barrett MS, Gallop R, Rynn MA, Rickels K. Short-term dynamic psychotherapy versus pharmacotherapy for major depressive disorder: a randomized, placebo-controlled trial. *J Clin Psychiatry* 2012; **73**: 66–73.

2

Barrett JE, Williams JW, Oxman TE, Frank E, Katon W, Sullivan M, *et al.* Treatment of dysthymia and minor depression in primary care: a randomized trial in patients aged 18 to 59 years. *J Fam Pract* 2001; **50**: 405–12.

3

Bedi N, Chilvers C, Churchill R, Dewey M, Duggan C, Fielding K, *et al.* Assessing effectiveness of treatment of depression in primary care. Partially randomised preference trial. *Br J Psychiatry* 2000; **177**: 312–8.

4

Blackburn IM, Moore RG. Controlled acute and follow-up trial of cognitive therapy and pharmacotherapy in out-patients with recurrent depression. *Br J Psychiatry* 1997; **171**: 328–34.

5

Blom MBJ, Jonker K, Dusseldorp E, Spinhoven P, Hoencamp E, Haffmans J, *et al.* Combination treatment for acute depression is superior only when psychotherapy is added to medication. *Psychother Psychosom* 2007; **76**: 289–97.

6

Browne G, Steiner M, Roberts J, Gafni A, Byrne C, Dunn E, *et al.* Sertraline and/or interpersonal psychotherapy for patients with dysthymic disorder in primary care: 6-month comparison with longitudinal 2-year follow-up of effectiveness and costs. *J Affect Disord* 2002; **68**: 317–30.

7

David D, Szentagotai A, Lupu V, Cosman D. Rational emotive behavior therapy, cognitive therapy, and medication in the treatment of major depressive disorder: a randomized clinical trial, posttreatment outcomes, and six-month follow-up. *J Clin Psychol* 2008; **64**: 728–46.

8

Dekker JJM, Koelen JA, Van HL, Schoevers RA, Peen J, Hendriksen M, *et al.* Speed of action: the relative efficacy of short psychodynamic supportive psychotherapy and pharmacotherapy in the first 8 weeks of a treatment algorithm for depression. *J Affect Disord* 2008; **109**: 183–8.

9

DeRubeis RJ, Hollon SD, Amsterdam JD, Shelton RC, Young PR, Salomon RM, *et al.* Cognitive therapy vs medications in the treatment of moderate to severe depression. *Arch Gen Psychiatry* 2005; **62**: 409–16.

10

Dimidjian S, Hollon SD, Dobson KS, Schmaling KB, Kohlenberg RJ, Addis ME, *et al.* Randomized trial of behavioral activation, cognitive therapy, and antidepressant medication in the acute treatment of adults with major depression. *J Consult Clin Psychol* 2006; **74**: 658–70.

11

Dunlop BW, Kelley ME, Mletzko TC, Velasquez CM, Craighead WE, Mayberg HS. Depression beliefs, treatment preference, and outcomes in a randomized trial for major depressive disorder. *J Psychiatr Res* 2012; **46**: 375–81.

12

Dunner DL, Schmaling KB, Hendrickson H, Becker J, Lehman A, Bea C. Cognitive therapy versus fluoxetine in the treatment of dysthymic disorder. *Depression* 1996; **4**: 34–41.

13

Elkin I, Shea MT, Watkins JT, Imber SD, Sotsky SM, Collins JF, *et al.* National Institute of Mental Health Treatment of Depression Collaborative Research Program. General effectiveness of treatments. *Arch Gen Psychiatry* 1989; **46**: 971–82; discussion 983.

14

Faramarzi M, Alipor A, Esmaelzadeh S, Kheirkhah F, Poladi K, Pash H. Treatment of depression and anxiety in infertile women: cognitive behavioral therapy versus fluoxetine. *J Affect Disord* 2008; **108**: 159–64.

15

Finkenzeller DW, Zobel I, Rietz S, Schramm E, Berger M. Interpersonelle Psychotherapie und Pharmakotherapie bei Post-Stroke-Depression. *Nervenarzt* 2009; **80**: 805–12.

16

Frank E, Cassano GB, Rucci P, Thompson WK, Kraemer HC, Fagiolini A, *et al.* Predictors and moderators of time to remission of major depression with interpersonal psychotherapy and SSRI pharmacotherapy. *Psychol Med* 2011; **41**: 151–62.

17

Hegerl U, Hautzinger M, Mergl R, Kohnen R, Schütze M, Scheunemann W, *et al.* Effects of pharmacotherapy and psychotherapy in depressed primary-care patients: a randomized, controlled trial including a patients’ choice arm. *Int J Neuropsychopharmacol* 2010; **13**: 31–44.

18

Hollon SD, DeRubeis RJ, Evans MD, Wiemer MJ, Garvey MJ, Grove WM, *et al.* Cognitive therapy and pharmacotherapy for depression. Singly and in combination. *Arch Gen Psychiatry* 1992; **49**: 774–81.

19

Jarrett RB, Schaffer M, McIntire D, Witt-Browder A, Kraft D, Risser RC. Treatment of atypical depression with cognitive therapy or phenelzine: a double-blind, placebo-controlled trial. *Arch Gen Psychiatry* 1999; **56**: 431–7.

20

Keller MB, McCullough JP, Klein DN, Arnow B, Dunner DL, Gelenberg AJ, *et al.* A Comparison of Nefazodone, the Cognitive Behavioral-Analysis System of Psychotherapy, and Their Combination for the Treatment of Chronic Depression. *New England Journal of Medicine* 2000; **342**: 1462–70.

21

Kennedy SH, Konarski JZ, Segal ZV, Lau MA, Bieling PJ, McIntyre RS, *et al.* Differences in brain glucose metabolism between responders to CBT and venlafaxine in a 16-week randomized controlled trial. *Am J Psychiatry* 2007; **164**: 778–88.

22

Markowitz JC, Kocsis JH, Bleiberg KL, Christos PJ, Sacks M. A comparative trial of psychotherapy and pharmacotherapy for ‘pure’ dysthymic patients. *J Affect Disord* 2005; **89**: 167–75.

23

Martin SD, Martin E, Rai SS, Richardson MA, Royall R. Brain blood flow changes in depressed patients treated with interpersonal psychotherapy or venlafaxine hydrochloride: preliminary findings. *Arch Gen Psychiatry* 2001; **58**: 641–8.

24

McKnight DL, Nelson-Gray RO, Barnhill J. Dexamethasone suppression test and response to cognitive therapy and antidepressant medication. *Behavior Therapy* 1992; **23**: 99–111.

25

McLean PD, Hakstian AR. Clinical depression: comparative efficacy of outpatient treatments. *J Consult Clin Psychol* 1979; **47**: 818–36.

26

Menchetti M, Rucci P, Bortolotti B, Bombi A, Scocco P, Kraemer HC, *et al.* Moderators of remission with interpersonal counselling or drug treatment in primary care patients with depression: randomised controlled trial. *Br J Psychiatry* 2014; **204**: 144–50.

27

Miranda J, Chung JY, Green BL, Krupnick J, Siddique J, Revicki DA, *et al.* Treating depression in predominantly low-income young minority women: a randomized controlled trial. *JAMA* 2003; **290**: 57–65.

28

Mohr DC, Boudewyn AC, Goodkin DE, Bostrom A, Epstein L. Comparative outcomes for individual cognitive-behavior therapy, supportive-expressive group psychotherapy, and sertraline for the treatment of depression in multiple sclerosis. *J Consult Clin Psychol* 2001; **69**: 942–9.

29

Moradveisi L, Huibers MJH, Renner F, Arasteh M, Arntz A. Behavioural activation v. antidepressant medication for treating depression in Iran: randomised trial. *Br J Psychiatry* 2013; **202**: 204–11.

30

Murphy GE, Simons AD, Wetzel RD, Lustman PJ. Cognitive therapy and pharmacotherapy. Singly and together in the treatment of depression. *Arch Gen Psychiatry* 1984; **41**: 33–41.

31

Mynors-Wallis LM, Gath DH, Day A, Baker F. Randomised controlled trial of problem solving treatment, antidepressant medication, and combined treatment for major depression in primary care. *BMJ* 2000; **320**: 26–30.

32

Mynors-Wallis LM, Gath DH, Lloyd-Thomas AR, Tomlinson D. Randomised controlled trial comparing problem solving treatment with amitriptyline and placebo for major depression in primary care. *BMJ* 1995; **310**: 441–5.

33

Parker G, Blanch B, Paterson A, Hadzi-Pavlovic D, Sheppard E, Manicavasagar V, *et al.* The superiority of antidepressant medication to cognitive behavior therapy in melancholic depressed patients: a 12-week single-blind randomized study. *Acta Psychiatr Scand* 2013; **128**: 271–81.

34

Quilty LC, McBride C, Bagby RM. Evidence for the cognitive mediational model of cognitive behavioural therapy for depression. *Psychol Med* 2008; **38**: 1531–41.

35

Rush AJ, Beck AT, Kovacs M, Hollon S. Comparative efficacy of cognitive therapy and pharmacotherapy in the treatment of depressed outpatients. *Cogn Ther Res* 1977; **1**: 17–37.

36

Salminen JK, Karlsson H, Hietala J, Kajander J, Aalto S, Markkula J, *et al.* Short-term psychodynamic psychotherapy and fluoxetine in major depressive disorder: a randomized comparative study. *Psychother Psychosom* 2008; **77**: 351–7.

37

Schulberg HC, Block MR, Madonia MJ, Scott CP, Rodriguez E, Imber SD, *et al.* Treating major depression in primary care practice. Eight-month clinical outcomes. *Arch Gen Psychiatry* 1996; **53**: 913–9.

38

Scott AI, Freeman CP. Edinburgh primary care depression study: treatment outcome, patient satisfaction, and cost after 16 weeks. *BMJ* 1992; **304**: 883–7.

39

Shamsaei F, Rahimi A, Zarabian MK, Sedehi M. Efficacy of Pharmacotherapy and Cognitive Therapy, Alone and in Combination in Major Depressive Disorder. *Hong Kong Journal of Psychiatry* 2008; **18**: 76.

40

Sharp DJ, Chew-Graham C, Tylee A, Lewis G, Howard L, Anderson I, *et al.* A pragmatic randomised controlled trial to compare antidepressants with a community-based psychosocial intervention for the treatment of women with postnatal depression: the RESPOND trial. *Health Technol Assess* 2010; **14**: iii – iv, ix – xi, 1–153.

41

Sloane RB, Staples FR, Schneider LS. Interpersonal therapy vs. nortriptyline for depression in the elderly. *Clinical and pharmacological studies in psychiatric disorders Biological psychiatry - new prospects*; **1985**: 344–6.

42

Thompson LW, Coon DW, Gallagher-Thompson D, Sommer BR, Koin D. Comparison of desipramine and cognitive/behavioral therapy in the treatment of elderly outpatients with mild-to-moderate depression. *Am J Geriatr Psychiatry* 2001; **9**: 225–40.

43

Weissman MM, Prusoff BA, Dimascio A, Neu C, Goklaney M, Klerman GL. The efficacy of drugs and psychotherapy in the treatment of acute depressive episodes. *Am J Psychiatry* 1979; **136**: 555–8.

44

Williams JW, Barrett J, Oxman T, Frank E, Katon W, Sullivan M, *et al.* Treatment of dysthymia and minor depression in primary care: A randomized controlled trial in older adults. *JAMA* 2000; **284**: 1519–26.

45

Zu S, Xiang Y-T, Liu J, Zhang L, Wang G, Ma X, *et al.* A comparison of cognitive-behavioral therapy, antidepressants, their combination and standard treatment for Chinese patients with moderate-severe major depressive disorders. *J Affect Disord* 2014; **152-154**: 262–7.
